# The Mediation Effects of Aluminum in Plasma and Dipeptidyl Peptidase Like Protein 6 (DPP6) Polymorphism on Renal Function via Genome-Wide Typing Association

**DOI:** 10.3390/ijerph181910484

**Published:** 2021-10-06

**Authors:** Ting-Hao Chen, Chen-Cheng Yang, Kuei-Hau Luo, Chia-Yen Dai, Yao-Chung Chuang, Hung-Yi Chuang

**Affiliations:** 1 Department of Public Health and Environmental Medicine, Research Center for Environmental Medicine, Kaohsiung Medical University, Kaohsiung 807, Taiwan; u105570007@kmu.edu.tw (T.-H.C.); u107800007@kmu.edu.tw (K.-H.L.); 2 Department of Occupational and Environmental Medicine, Kaohsiung Medical University Hospital, Kaohsiung 807, Taiwan; u106800001@kmu.edu.tw (C.-C.Y.); daichiayen@gmail.com (C.-Y.D.); 3 Department of Neurology, Kaohsiung Chang Gung Memorial Hospital, Kaohsiung 833, Taiwan; ycchuang@adm.cgmh.org.tw

**Keywords:** aluminum, Taiwan Biobank, estimated glomerular filtration rate, genome-wide association, mediation analysis, dipeptidyl peptidase-like protein 6 (DPP6) gene

## Abstract

Aluminum (Al) toxicity is related to renal failure and the failure of other systems. Although there were some genome-wide association studies (GWAS) in Australia and England, there were no GWAS about Han Chinese to our knowledge. Thus, this research focused on using whole genomic genotypes from the Taiwan Biobank for exploring the association between Al concentrations in plasma and renal function. Participants, who underwent questionnaire interviews, biomarkers, and genotyping, were from the Taiwan Biobank database. Then, we measured their plasma Al concentrations with ICP-MS in the laboratory at Kaohsiung Medical University. We used this data to link genome-wide association (GWA) tests while looking for candidate genes and associated plasma Al concentration to renal function. Furthermore, we examined the path relationship between Single Nucleotide Polymorphisms (SNPs), Al concentrations, and estimated glomerular filtration rates (eGFR) through the mediation analysis with 3000 replication bootstraps. Following the principles of GWAS, we focused on three SNPs within the dipeptidyl peptidase-like protein 6 (*DPP6*) gene in chromosome 7, rs10224371, rs2316242, and rs10268004, respectively. The results of the mediation analysis showed that all of the selected SNPs have indirectly affected eGFR through a mediation of Al concentrations. Our analysis revealed the association between *DPP6* SNPs, plasma Al concentrations, and eGFR. However, further longitudinal studies and research on mechanism are in need. Our analysis was still be the first study that explored the association between the *DPP6*, SNPs, and Al in plasma affecting eGFR.

## 1. Introduction

Aluminum (Al), which is the third most common element on earth, is widely used in many daily necessities and energy development [1]. Even daily drinking water may contain a little bit of Al. However, as industries advance, humans demand more Al, and Al-related occupational diseases have increased gradually [2,3,4]. Al is an unnecessary element for the human body. There are many studies that indicate that Al toxicity would damage renal function [5,6] and other systems in humans [7,8,9,10]. Al is excluded through 95% kidney and 2% bile. However, Al toxicity and decline of renal function were associated with each other. Patients with renal failure were damaged by Al. On the other hand, chronic kidney disease decreased Al excretion and accumulated it in the human body. The metabolism of Al in the human body is not clear; however, some enzymes and genotypes have been studied [11].

A genome-wide association study showed that the most significant SNP for Al in serum was at chr8:9095620, which is near *PPP1R3B*, but the biological information of this SNP was unknown [11]. Moreover, an occupational epidemiology study indicated that exposure to metal particles could cause DNA methylation; in an unadjusted model, exposure to Al particles was associated with DNA methylation in tumor suppressor genes; however, after adjustment for smoking, age, and body mass index (BMI), Al was not associated with DNA methylation [12]. Some epidemiological studies indicated that there was no association between Al exposure and Long Interspersed Element-1 (LINE-1) methylation [13,14]. In addition, a Chinese study group investigated whether those with occupational exposure to Al would have mild cognitive dysfunction and global DNA methylation [15], which causes a significant decrease in global DNA methylation. Another occupational epidemiological study from China indicated that occupational exposure to Al would have lowered the Amyloid Precursor Protein (APP) gene-methylation [15]. On the other hand, gene expression had an important role in influencing the maximum tolerance of Al toxicity [16], and different single nucleotide polymorphisms (SNPs) also affected different gene expressions in an animal model [11,17]. To our knowledge, there is no relevant study about the Han Chinese population.

Using the genome-wide data of Taiwan’s Han population, we could provide some evidence to look for potentially susceptible SNPs of a southern Chinese population to Al toxicity and understand the relationship between Al levels and genetic variants. Therefore, the goal of this study was to explore potential SNPs that involve plasma Al-affected renal function.

## 2. Materials and Methods

### 2.1. Study Population

The subjects of this cross-sectional study consisted of 500 Taiwan Han Chinese subjects from Taiwan Biobanks [18,19], included 30–70-year-old healthy people and excluded those diagnosed with cancer, non-Taiwanese people, and patients who were hospitalized from inception to date. The purpose of the Taiwan Biobank data is to establish the baseline characteristics of the Taiwan population based on volunteers who genuinely cared about their own health status. The Taiwan Biobank consisted of a population-based databank and storage-plasma bank. The study was conducted according to the guidelines of the Declaration of Helsinki and approved by the Institutional Review Board of Kaohsiung Medical University Hospital (KMU-HIRB-E(I)-20150259, the initial date of approval: 6 January 2016). Approval was waived for individual consent forms due to de-identification in Taiwan Biobank data and specimens.

### 2.2. Questionnaires

Each subject was administered questionnaires about demographic information, personal health behavior, present medication, drug use, diet, female-related questions, family history, lifestyle, economic status, and personalized information about the living environment.

### 2.3. Laboratory Analysis

Biomarkers of the Taiwan Biobank were all analyzed in the Linkou Chang Gung Memorial Hospital. However, the plasma Al concentrations were measured in the laboratory at Kaohsiung Medical University by an inductively coupled plasma mass spectrometry (ICP-MS, Thermo Scientific XSERIES 2^®^, Waltham, MA USA). The resolution was set to 0.8 and 0.4 amu at 10% peak height, which complied with the typical Al analysis. Radio Corporation of America (RCA) cleaning standard was used on all equipment used in the laboratory. For sample preparation, 1% HNO3 was added to the plasma samples to make a 1:10 dilution, and then we waited for 10 min. For checking the high linearity, ICP-MS calibration standard solution (from Accu Standard, MES-04-1) was diluted to 0.1, 0.2, 0.5, 1, 2, 5, 10, 20, 50, 100, 200, 500, 1000, 2000, and 3000 ug/L to estimate the calibration curve, which showed a high correlation coefficient (r > 0.995). Before analyzing the unknown concentrations, we conducted quality assurance (QA) and quality control (QC) to ensure precision and accuracy. QA was used to analyze standard reference materials (SRMs). To ensure consistency of the laboratory test, we took random SRMs to conduct repeated analyses. Each result had to fit the reference between 90% and 110%. QC was to ensure the stability of the system by triple testing the SRM sample, of which the coefficient of variance (CV) should be less than 3%.

### 2.4. Genotyping

DNA samples were genotyped by a customized Axiom Taiwan Biobank Array Plate (Taiwan Biobank chip, TWB chip) to obtain 645,918 single-nucleotide polymorphisms (SNP). The TWB chip was based on Affymetrix databases, the HapMap Project [20], the 1000-Genome Project [21,22], and published GWAS research about the Han Chinese population in Beijing and Taiwan to pick up SNPs related to Taiwan’s Han population.

To increase quality control in the genotype data, we looked for the subjects with high missing genotyping rates and extreme heterozygosity rates. The sample was excluded if the missing genotype rate was >5%, and SNP were excluded if the missing rate was >5%. We used (N-O)/N to calculate the mean heterozygosity rate, where N was the number of non-missing genotypes and O was the observed number of homozygous genotypes. Individuals with more than 3 standard deviations from the mean heterozygosity rate indicated that the DNA sample was contaminated or inbred; therefore, we excluded the subjects with extreme heterozygosity rates. We estimated the exact *p*-value of the Hardy–Weinberg equilibrium (HWE), and SNPs were excluded if the *p*-value of HWE was < 10^−6^. Furthermore, we calculated identity by descent (IBD). To understand whether our sample was independent, we excluded individuals if IBD was > 0.1875. All quality controls were conducted using PLINK software (version 1.9) [23] and R language (version 3.4.1) [24].

### 2.5. Statistical Analysis

#### 2.5.1. Descriptive Analysis

We used the mean ± SD and quartiles to represent continuous data and n (%) to represent categorical data. Blood measurements were log-transformed to generate normal distribution such as Al concentration. The MDRD equation was used to evaluate renal function [25]:

eGFR = 175• (Serum creatinine)^−1.154^ • (Age)^−0.203^ • 0.742 (if female)• 1.21 (if African American) (1)

We used eGFR as the *x*-axis and Al in plasma as the *y*-axis to make the scatter plot with a regression line to understand the distribution of eGFR and Al levels (log-transformed) in plasma. All descriptive analyses were conducted with SAS software (version 9.4).

#### 2.5.2. Genome-Wide Association (GWA)

Association testing for log-transformed Al was conducted in the linear regressions through an additive model: SNP types were coded by assigning 0, 1, and 2 for homozygous genotypes with 0 minor alleles, heterozygous genotypes with 1 minor allele, and homozygous genotypes with 2 minor alleles, respectively.

We performed the crude model for log-transformed Al to avoid Type I errors because of multiple comparisons. The Bonferroni Correction [26] was used to correct the *p*-value of multiple testing. The corrected *p*-value = 0.05/numbers of SNPs, therefore the corrected *p*-value was (0.05/645918) = 8 × 10^−8^ and the suggested p-value was 1 ×∙10^−6^. In addition, a Manhattan plot was used to visualize the results of association testing. The vertical axis was −log_10_ (*p*-value), where a higher location meant a smaller p-value, and the horizontal axis represented the numbers of the chromosomes, and each dot represented each SNP. Meanwhile, we generated a QQ-plot to check the quality of the GWA results, where the *Y*-axis represented the observed −log_10_ (*p*-value), *X*-axis meant the expected −log_10_ (*p*-value), and each dot represented each SNP. We used g-profiler [27] to finish gene annotation.

All association tests were performed with PLINK 1.9 [23], and R statistical software (version 3.4.1) [24]. The Manhattan plot was conducted by Haploview software [28,29].

#### 2.5.3. Mediation Analysis

**Figure 1:**
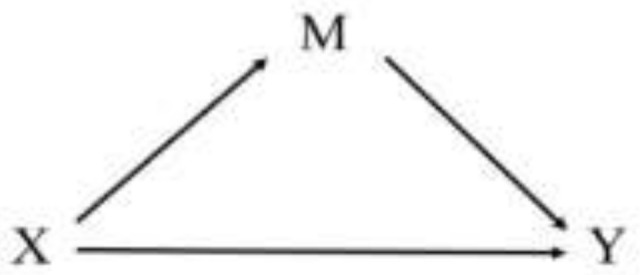
The relationship between X (e.g., SNPs), M (e.g., Al concentrations), and Y (e.g., eGFR) using a mediation analysis.

We performed a mediation analysis to examine if there was an indirect effect between SNPs and renal function (Figure 1).

According to the assumption of Baron et al. [30], there were three criteria that had to be met: first of all was the exposure to outcome association; the second was exposure to mediator association; the third was the mediator to outcome association. However, MacKinnon et al. indicated that even it did not follow the first assumption of Baron, there was still mediation [31]. Hence, we built three linear models to test whether to follow the assumption of Baron.

Y = b + b_1_ X_1_ + b_2_ X_2_ + ….+biXi (2)

M = a + a_1_ X_1_ (3)

Y = r + r_x_X_i_ + r_m_M_i_ (4)

To generate the normalized residual, we used log-transformed Al concentration and assigned 0, 1, and 2 for homozygous genotypes, heterozygous genotypes, and homozygous genotypes with 2 minor alleles to fit an additive model. The causal mediated effect was estimated using the product-of-coefficient method to generate the mediation coefficient [31,32,33,34].

We used the bootstrap method to estimate the indirect effect because bootstrap CIs did not require the assumption of normal distribution and did not require the sample size to be too large [35,36]. We used percentile bootstrap CIs to estimate the confidence interval for avoiding the type-I error. All mediation analyses were conducted with STATA 13.0 software, and a *p*-value <0.05 was considered statistically significant.

## 3. Results

### 3.1. Study Population and SNPs Typing

**Figure 2:**
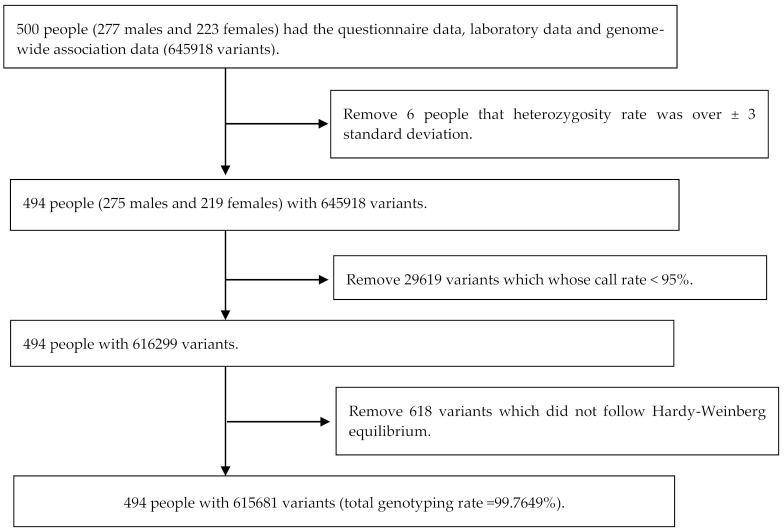
Flow chart for quality control of SNPs and selection of study population.

After collecting the questionnaires and blood samples, 500 subjects were involved in this study; however, there was a subject who rejected to participate in this study. In the beginning, there were 645,918 SNPs in the Taiwan Biobank chip (TWB chip) and 500 subjects (227 males and 223 females). As illustrated in Figure 1, there were 6 people whose heterozygosity rate was over ± 3 standard deviations and 29,619 variants whose call rate was <95%. There were 618 variants that did not follow the Hardy–Weinberg equilibrium. Finally, we conducted a further analysis with 494 subjects and 615,681 variants in which the total genotyping rate was 99.7649% (Figure 2).

### 3.2. Characteristics of Study Population

The demographic characteristics of the study population are shown in Table 1. The average age was 48.28 years old, the rate of people who had drunk for more than six months was about 7.9%, and the percentage of people who had smoking experience was 34.21%. Regarding the indication of renal function, blood urine nitrogen (BUN), creatinine, and uric acid (UA) were measured. Among 75% of participants who were measured, BUN was under 15.20 mg/dL. A total of 75% of subjects were under 0.91 mg/dL, and 75% of subjects were under 6.9 mg/dL, respectively. The mean (SD) values for BUN, creatinine, and UA were 13.06 (3.48), 0.78 (0.19), and 5.73 (1.56), respectively. Using creatinine levels to estimate the eGFR, the mean (SD) was 99.52 (22.00), and almost 75% of subjects were >85.03. As for the blood measurement, the mean (SD) values for HbA1c (%) were 5.66 (0.55). Almost 75% of the participants were under the normal range in blood measurement. In the indicator of total cholesterol, high-density lipoprotein (HDL), low-density lipoprotein (LDL), and liver function (AST and ALT), the mean (SD) values were 197.72 (35.75), 53.79 (13.19), 125.52 (32.42), 23.38 (11.44), and 25.56 (23.36), respectively, which showed this was a relatively healthy population base. Regarding the Al concentration in plasma (μg/L), the mean value was 1.13 ± 0.80 (Table 1).

### 3.3. Association between eGFR and Al

**Figure 3:**
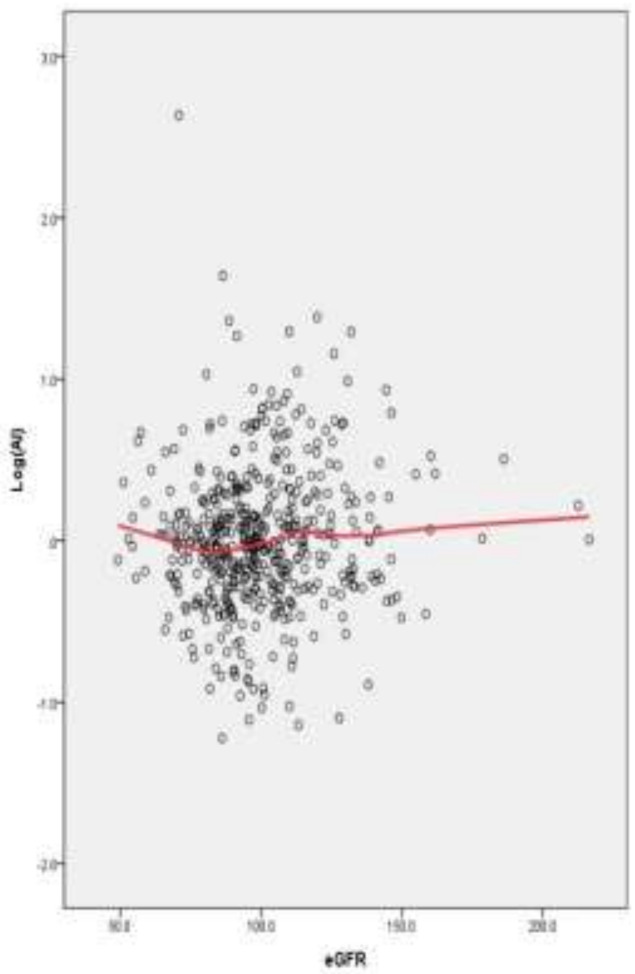
Scatter plot for eGFR and log-transformed Al concentration.

Figure 3 showed the association between eGFR and Al (log-transformed). We used a smoothing curve (smooth function: loess, 65% of point to fit, df = 4, kernel: Epanechkinov) to reveal that eGFRs ranged normal (between 85 and 120) and had lower plasma Al levels.

### 3.4. Genome-Wide Association Test

**Figure 4:**
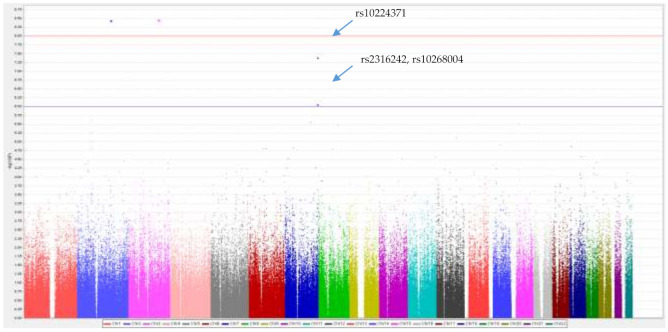
Manhattan plot for plasma aluminum after controlling for gender, age, smoking, and alcohol consumption. The vertical axis was −log_10_ (*p*-value) according to the strength of association. The horizontal axis represents chromosome location according to the genomic location. The red line represents significant levels (*p*-value < 8 × 10^−8^). The blue line represents suggestive levels (*p*-value < 1 × 10^−6^).

We did not use the SNPs of the Y chromosome. In Figure 4, there were two loci beyond the significant level (*p*-value < 8 × 10^−8^) and three variants between the significant level and suggestive level (*p*-value < 1 × 10^−6^) in the genome-wide association test. The most significant SNP was rs9857275, which was an intron variant located in *ZBTB38 (zinc finger and BTB domain containing 38)*, a protein encoded by this gene was associated with DNA methylation [37]. The second was rs764111946, which was a missense variant and found in chromosome 2, showed close association to the *BAZ2B (bromodomain adjacent to zinc finger domain 2B)* gene [38]. Within (or close to) the *dipeptidyl peptidase-like protein 6 (DPP6) gene* were three intron variants that were at the suggestive level (*p* < 10^−6^). However, the minor allele frequencies of SNPs (rs9857275, rs764111946) were too small (both MAF = 0.001), and the first 2 SNPs beyond the significant level were so rare that it was difficult to estimate the true effect of the rare variants based on the current sample size (minor allele frequency = 0.001). Hence, we focused on the variants with bigger MAF, and we decided to focus on *DPP6* variants (rs10224371, rs2316242, rs10268004) using a medication analysis.

### 3.5. Mediation Analysis

#### 3.5.1. rs10224371→ln(Al)→eGFR

**Figure 5:**
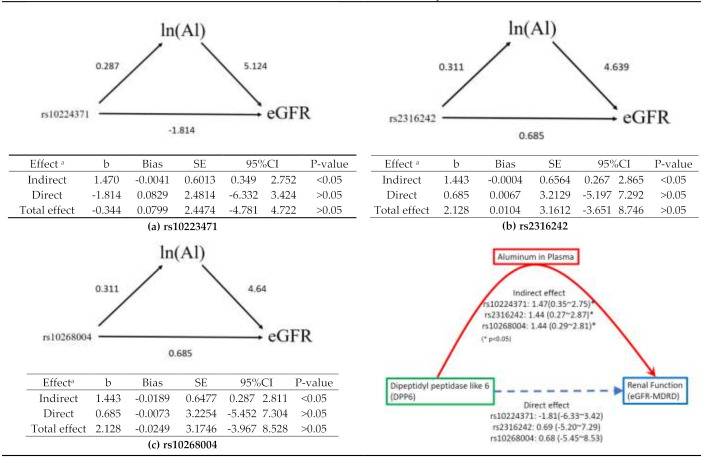
The mediation analysis of 3 SNPs within DPP6, ln (aluminum) levels in plasma, and eGFR. (**a**): rs10224371 was fitted as an additive model effect with 0, 1, and 2 for CC, CT, and TT. (**b**): rs2316242 was fitted as an additive model effect with 0, 1, and 2 for CC, CT, and TT. (**c**): rs10268004 was fitted as an additive model effect with 0, 1, and 2 for TT, GT, and GG. (a): each effect with adjusted gender, age, smoke experience, and drinking habits. (**b**): coefficient; SE, standard error; 95%CI, 95% confidence interval estimated by percentile bootstrap methods. A summary is shown in the right lower quadrant. * *p* < 0.05.

As shown in Figure 5a, rs10223471, which was located in *DPP6*, was significantly correlated with ln(Al)(coefficient = 0.287, 95%CI = 0.186, 0.387) after controlling for potential confounders, and ln(Al) was correlated with eGFR (coefficient = 5.124, 95%CI = 0.971, 9.277). The evidence indicated that rs10223471 within *DPP6* would lean to increased eGFR through a mediated effect of ln(Al) (coefficient = 1469, 95%CI = 0.349, 2.751); however, the direct effect was non-significant (coefficient = −1.814, 95%CI = −6.332, 3.424). This polymorphism only had an indirect effect. what it meant was that there was to for the exposure to Al which thus resulted in increased eGFR.

#### 3.5.2. rs2316242→ln(Al)→eGFR

As shown in Figure 5b, the first path (rs2316242→ln(Al)) showed that ln(Al) would increase by 0.311 units when a minor T allele of rs2316242 existed, and ln(Al) would increase eGFR by 4.639 units (95%CI = 0.509, 8.768). A bootstrap with 3000 replications showed that every T allele of rs2316242 would cause an increase in eGFR by 1.443 units (95%CI = 0.320, 2.936) through a mediated effect of ln(Al). However, rs2313242 did not result in an increased eGFR directly (coefficient = 0.6848, 95%CI = −4.995, 7.446).

#### 3.5.3. rs10268004→ln(Al)→eGFR

As shown in Figure 5c, rs10268004, within *DPP6*, would cause a significant increase in ln(Al) by 0.311 units (95%CI = 0.189, 0.433), and a unit of increase in ln(Al) would cause eGFR to increase by 4.64 units (95%CI = 0.509, 8.768). A bootstrap with 3000 replications suggested that rs10268004 would result in increased eGFR by 1.443 units (95%CI = 0.379, 2.964) through a mediated effect of ln(Al). However, no direct evidence indicated that rs10268004 could result in increased eGFR directly.

## 4. Discussion

Some studies have been based on subjects who had occupational exposure to Al [2,3,4], while in our study, subjects were from the Taiwanese population. Al is an unnecessary element for the human body. There were probably some differences that affected the element’s levels in blood, such as dietary habits, local customs, or ethnic differences. In addition, the element’s levels in whole blood, serum, or plasma were different, while too many impurities in the whole blood would cause the test results to be unstable; moreover, the differences between serum and plasma we34re the proteins that would bind with some special metal such as lead, mercury, arsenic, and nickel [39]. Al levels in plasma could be consistent with the reference value from France [40], which shows that there was no occupational exposure to Al. Thus, this research could be a representation of plasma Al levels associated with renal function in the Taiwanese population.

A GWAS technique was used to find out the potential SNPs of the interested phenotype; therefore, we used plasma Al levels as a phenotype to perform the association analysis. There were three SNPs found in the same linkage disequilibrium (LD) blocks, rs10224371, rs2316242, and rs10268004, within *DPP6*. This gene, *DPP6*, has been proven as a candidate gene for amyotrophic lateral sclerosis (ALS) in the patients with ALS against the non-ALS population [41,42,43] and could also be a potential factor that led to mental diseases, such as autism [44] and posttraumatic stress disorder (PTSD) [45]. Therefore, the protein encoded by *DPP6* is associated with the regulation of the potassium ion transport channel and further causes neurodevelopmental impairment [46,47] and mental diseases [44,45]. In our findings, there were three SNPs within *DPP6* with suggestively significant association levels of Al concentration in plasma. Based on the biological evidence, Al may replace iron ions and calcium ions in the brain and interfere with other trace elements [48], and the protein encoded by *DPP6* regulates the potassium ion transport channel and causes abnormal synapse activity [46,47]. Esther Ng et al. found a potential variant within chr8:9095620, which was reported at *PPP1R3B*, and three SNPs were found in chr7:89168136, chr7:73882011, and chr7:136945107, which report genes located at *ZNF804B*, *GTF2IRD1*, and *PTN*. The differences in our findings were probably due to race and type of experimental sample; Esther Ng et al. used the serum to measure the Al levels, and the study population was from Uppsala, Sweden [11]. There are more than one-third of genome differences between the Han Chinese population and Caucasians. This may be the main reason that leads to inconsistencies between Esther Ng et al. and our study [11].

The previous studies indicated that metal toxicity caused renal tubular damage [39,49] and makes the body unable to excrete the metal. However, according to some studies, the ability of Al excretion for patients with renal failure was worse than the ability of the normal population [50]. In our research, the majority of the study population were volunteers who greatly care about their own health status. As illustrated in Figure 3, a U-shaped curve existed. When eGFR was between 90 and 110, Al levels in plasma were the lowest; however, this U-shaped curve supports the evidence that previous studies indicated [50].

In addition, the indirect effect of *DPP6* was observed. This represented that *DPP6* would have no effect on eGFR if there was no Al in human plasma. This proved that *DPP6* was associated with some neurological diseases, including cognitive diseases and memory impairment [41,42,43,44,45,47,51]. Some studies also mentioned that exposure to Al would be a potential factor in the development of Alzheimer’s disease, but the causal relationship was still controversial [52,53,54,55]; however, our finding showed that there was an association between Al in plasma, *DPP6*, and eGFR. To our knowledge, there were few studies that explained the indirect effect of *DPP6* on eGFR. Moreover, in our findings, three variants in *DPP6* were within the same LD-blocks. Therefore, it may be interesting to further investigate the probable causal effect through deep sequencing and the gene expression of *DPP6* on renal function through molecular biology experiments.

The first limitation of this study was its relatively small sample size. To detect the rare variant with enough statistical power, increasing the sample size is needed. Meanwhile, statistical power would be limited because of multiple-testing correction; therefore, only large-scale gene-versus-phenotype effects can be discovered. The second limitation was the study design. Since a cross-sectional study could not ensure a causal relationship, longer follow-up data is in need to evaluate the long-term impact of Al levels on renal function. Eventually, due to the low exposure to Al in the normal population, the data from occupationally exposed workers will provide more information for this kind of study.

## 5. Conclusions

To the best of our knowledge, this is the first GWA study to explore the association between candidate variants and Al concentration in plasma on the Han Chinese population. Furthermore, we found that *DPP6* variants played a mediator between Al in plasma and renal function. Though, longitudinal data or data of occupational exposure are in need. Nevertheless, these findings can provide additional information and medical care for the decision making of occupational medicine.

## Figures and Tables

**Table 1:** Characteristics of study population.

Term (*n* = 494)	Mean ± SD, N(%)	Min	Q1	Q2	Q3	Max
Gender (Male)	275(55.7%)					
Drink (Current drinking) ^a^	39(7.9%)					
Smoke experience (Yes)	169(34.2%)					
Age (year)	48.3 ± 10.9	30	40	47	54	70
Height (cm)	165.18 ± 8.63	142.0	188.5	158.5	165.5	171.5
Weight (kg)	66.95 ± 12.32	39.4	120.8	58.2	66.80	74.8
γ-GT (U/L)	26.25 ± 27.09	6.0	13.0	18.0	30.0	293.0
BUN (mg/dL)	13.06 ± 3.48	5.0	10.6	12.6	15.2	28.2
Creatinine (mg/dL)	0.78 ± 0.19	0.33	0.62	0.79	0.91	1.50
eGFR ^b^ (mL/min/1.73 m^2^)	99.52 ± 22.00	49.15	85.30	96.99	110.37	216.66
Uric acid (mg/dL)	5.73 ± 1.56	2.6	4.5	5.6	6.9	12.4
HbA1C (%)	5.66 ± 0.55	4.4	5.4	5.6	5.8	11.9
Total cholesterol (mg/dL)	197.7 ± 35.8	117	358	174	195	217
TG (mg/dL)	122.9 ± 85.7	30	798	68	101	145
HDL (mg/dL)	53.8 ± 13.2	26	108	44	52	62
LDL (mg/dL)	125.5 ± 32.4	47	258	105	124	146
AST (U/L)	23.38 ± 11.44	10	18	21	26	167
ALT (U/L)	25.6 ± 23.4	7	15	20	28	344
Aluminum (μg/L)	1.13 ± 0.80	0.29	0.78	0.99	1.27	13.94

^a^ To continue alcohol consumption for more than six months, 155 cc per week. ^b^ Estimated glomerular filtration rate (MDRD equation); Q1: The first quartile; Q2: The second quartile; Q3: The third quartile.

## Data Availability

The data from Taiwan Biobank in this study can be applied from the Taiwan Biobank at https://www.twbiobank.org.tw/new_web_en/about-export.php (accessed on 20 August 2021).
